# Utilizing Bioinformatics Technology to Explore the Potential Mechanism of Danggui Buxue Decoction against NSCLC

**DOI:** 10.1155/2022/5296830

**Published:** 2022-02-26

**Authors:** Bin Yu, Guangyao Lv, Muhammad Sohail, Zhiyong Li, Yanli Li, Meiyu Yu, Fuyou Sun, Hui Xu

**Affiliations:** ^1^School of Pharmacy, Collaborative Innovation Center of Advanced Drug Delivery System and Biotech Drugs in Universities of Shandong, Key Laboratory of Molecular Pharmacology and Drug Evaluation, Yantai University, Yantai, Shandong Province, China; ^2^Guangdong Zhongrun Pharmaceutical R&D Co., Ltd., Guangzhou, Guangdong Province, China; ^3^Department of Pharmaceutics, Yantai Muping Hospital of Traditional Chinese Medicine, Yantai, Shandong Province, China

## Abstract

While lung cancer poses a serious threat to human health, non-small-cell lung cancer (NSCLC) is the most common type of lung cancer. Danggui Buxue Decoction (DBD) is a classical traditional antitumor medicine commonly used in China. However, the potential mechanism of DBD against NSCLC has not yet been expounded. Therefore, this study clarified the potential molecular mechanism and key targets of DBD in NSCLC treatment through several technological advances, such as network pharmacology, molecular docking, and bioinformatics. Firstly, the relative active ingredients and key DBD targets were analyzed, and subsequently, a drug-ingredient-target-disease network diagram was constructed for NSCLC treatment with DBD, resulting in the identification of five main active ingredients and ten core targets according to the enrichment degree. The enrichment analysis revealed that DBD can achieve the purpose of treating NSCLC through the AGE-RAGE signaling pathway in diabetic complications. Secondly, the molecular docking approach predicted that quercetin and hederagenin have the best working mechanisms with PDE3A and PTGS1, while the survival analysis results depicted that high PDE3A gene expression has a relatively poor prognosis for NSCLC patients (*p* < 0.05). Additionally, PDE3A is mainly distributed in the LU65 cell line that originated from Asian population. In summary, our study results showed that DBD can treat NSCLC through the synergistic correlation between multiple ingredients, multiple targets, and multiple pathways, thus effectively improving NSCLC prognosis. This study not only reflected the medicinal value of DBD but also provided a solid structural basis for future new drug developments and targeted therapies.

## 1. Introduction

According to the estimates from World Health Organization's “Global Cancer Report 2020,” lung cancer (2.21 million cases) remains the second most commonly diagnosed cancer in the world [1]. The report also predicts that by 2040, due to the incidence rate being 20%, the number of new cancer cases will exceed 27 million worldwide, with one in every five people suffering from cancer. In 2020, an estimated 4.57 million new cancer cases were reported in China, of which lung cancer is by far the leading cause of the highest number of reported cases at 23.7% and is still the most frequently occurring cancer threatening the health of the Chinese people [1–3]. Among all the lung cancer subtypes, NSCLC is the most common form, accounting for 80% of all lung cancer diagnoses with a 5-year survival rate of 18% [4, 5]. As the NSCLC mortality rate is reaching towering proportions every year [6], the conventional treatment regime is still based on chemotherapy or radiotherapy, which predicts a poorer prognosis, reduces the quality of life, and increases the economic burden on patients and their families through expensive treatment costs [7]. Although the emergence of targeted therapy is now being considered a boon for cancer patients, the resultant increase in drug resistance and unexplained incidence of allergic reactions make it inappropriate for some patients [8, 9]. In the present scenario, several other studies involved in research on traditional Chinese medicine (TCM) have also postulated that TCM has its unique advantages while treating tumors with significant curative effects, fewer side effects, and low drug resistance and can also manifest excellent outcomes in combination with other conventional drugs [10–13].

As described in Li Gao's “Differentiation of Endogenous and Exogenous Disorders,” DBD is a fixed-ratio (5 : 1) coformulation consisting of Chinese medicines Danggui and Huangqi, which is also considered as a classic formula for invigorating qi and producing blood, thus increasing hematopoietic function [14]. Danggui is the dried root of a perennial leguminous plant Astragalus membranaceus (Fisch.) Bge. var. mongholicus (Bge.) Hsiao (AMB) [15] whereas Huangqi is the dry root of Angelica sinensis (Oliv.) Diels (family Umbellaceae) [16]. A plethora of pharmacological studies revealed that DBD regulates immune responses [17], promotes hematopoiesis [17, 18], protects the cardiovascular system [19], shields the liver [20], and demonstrates antitumor properties [21]. DBD has a good clinical effect on NSCLC and can effectively improve the quality of life of patients with advanced NSCLC; when used alone or in combination with other conventional drugs, it can also reduce the side effects of radiotherapy and chemotherapy as well as improve their sensitivity [22–25]. Although it was observed in clinical practice that DBD is highly effective in the NSCLC treatment, the complex composition of DBD and the unclear mechanism of action make it quite difficult to conduct further research. Henceforth, TCM being a traditional medicine can also treat diseases through multicomponent, multitarget, and multipathway synergistic effects to achieve the purpose of curative healing [26]. However, the interconnected complexity of traditional Chinese medicine components and their indistinct mechanism of action duly limit further development and usage of such traditional medicines [27].

As a novel research approach, network pharmacology is based on high-throughput omics data analysis, computer virtual computing, and network database retrieval, which acts as an effective means for discovering active drug substances and revealing their pharmacological mechanisms of action from a crucial perspective of molecular biological networks [28]. The multicomponent, multitarget, and multipathway characteristics of Chinese herbal compound prescriptions are the prime focus domain of network pharmacology research [29]. Although molecular docking is mainly used to study the interaction between the molecules and predict salient binding modes and relationships, it is also a mathematical simulation of drug-receptor interactions to predict the most active drug component [30].

**Figure 1:**
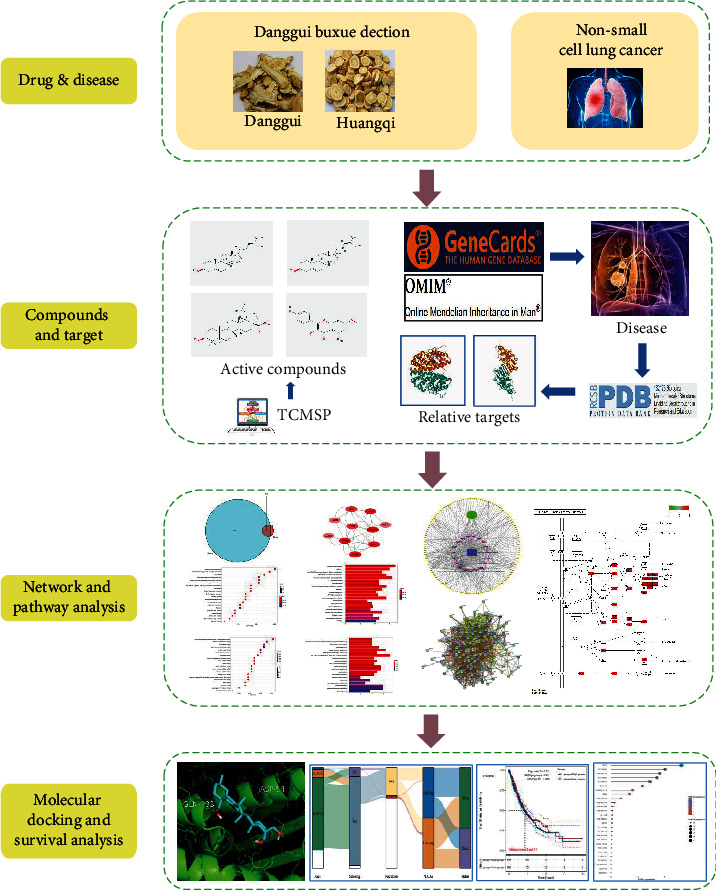
Technological plan for this study.

Owing to the fact that there is no network pharmacology study in the literature to date on NSCLC treatment with DBD, this study explored the functional molecular mechanism of DBD in NSCLC treatment through network pharmacology and molecular docking as well as analyzed the survival of important targets through The Cancer Genome Atlas (TCGA) database to duly assess the target's impact on NSCLC prognosis. In addition, this study also conducted an in-depth analysis of the expression of important targets in NSCLC tumor cell lines through the Cancer Cell Line Encyclopedia (CCLE) database. Our research plan is shown in Figure 1.

## 2. Materials and Methods

### 2.1. Collection of Active Ingredients and Chemical Structure of DBD

Traditional Chinese medicine systems pharmacology (TCMSP) database (https://tcmsp-e.com/) was used to collect the active ingredients of DBD. For identifying the active ingredients in DSD, “Danggui” was heavily explored in the database, followed by scheduling screening conditions through oral bioavailability (OB) and druglikeness (DL) parameters to acquire the needed active ingredients and downloading them. Since OB represents the percentage of traditional Chinese medicine's absorption in the human circulatory system, while DL is the similarity between the ingredients and known drugs [31], the following values (OB > 30% and DL > 0.18) were decided as the foremost screening conditions of all active ingredients. “Huangqi” was also profoundly searched through the TCMSP database and was filtered and downloaded according to these values (OB > 30% and DL > 0.18) set as the screening conditions.

The TCMSP database contains the chemical structure of all the active ingredients. The corresponding chemical structure of obtained active ingredients can also be downloaded through the TCMSP database. Additionally, active ingredients' Tripos molecule structure format (MOL2) files were also obtained for further utilization in the molecular docking procedure. Due to the nonavailability of information about the main active ingredients, MOL2 files of all active ingredients were downloaded for further screening processes. However, in the case of active ingredients lacking MOL2 files, ChemBioDraw Ultra and ChemBio3D Ultra software were used to draw them and later saved them in MOL2 format for further usage.

### 2.2. The Potential Target Database of NSCLC

We used the GeneCards database (https://www.genecards.org/) and Online Mendelian Inheritance in Man (OMIM database) (https://omim.org/) to search the NSCLC-related targets. For instance, after inserting “Non-Small Cell Lung Cancer” as a keyword in two databases, the relevant targets downloaded in databases were merged and then deduplicated to acquire the NSCLC relevant targets. After the utilization of R software to draw a Venn diagram of DBD and NSCLC targets, finally, our core targets were achieved.

### 2.3. Protein-Protein Interaction Construction

The construction of the protein-protein interaction (PPI) network was completed on the string (https://string-db.org/) website. Firstly, the “Multiple Proteins” option was selected, followed by mentioning the core targets in the List of Names and inserting “homo sapiens” in the Organism inquiry. Lastly, after inserting the default values for other parameters, the PPI network map was obtained for our perusal.

### 2.4. Drug-Ingredient-Target-Disease Network Construction

A Cytoscape software was employed to build a drug-ingredient-target-disease visualization network diagram. After inserting the obtained drugs, ingredients, core targets, diseases, and their mutual correspondence mechanisms into the Cytoscape software, the topological characteristics of network nodes were calculated through the Cytoscape plugin Network Analyzer that included various degrees of correlation, namely, degree, betweenness, and closeness, among which the degree value was an important evaluation index [32]. Subsequently, ten main targets were also acquired through the degree correlation factor of the Cytoscape software.

### 2.5. GO Functional Analysis and KEGG Enrichment Pathway Analysis

In order to further understand the function of the core targets and the key DBD pathway against NSCLC, a Gene Ontology (GO) functional enrichment analysis and Kyoto Encyclopedia of Genes and Genomes (KEGG) enrichment pathway analysis were conducted. GO functional enrichment analysis was performed by using Cytoscape software and R software while the results consisted of an amalgamation of biological process, cellular component, and molecular function and were presented in a bubble chart and a histogram with a *p* value where *p* < 0.05 indicated statistical significance. KEGG enrichment pathway analysis was also performed through Cytoscape software and R software, while their results included a bubble chart, a histogram, and some signal path diagrams with a *p* value; *p* < 0.05 indicated statistical significance.

### 2.6. Molecular Docking

AutoDock Vina software was used to verify the molecular docking mechanism between the top ten key targets in the PPI network and the five main active DBD ingredients. The three-dimensional structure of the ten target proteins from the RCSB PDB protein structure database (https://www.rcsb.org/) and the chemical structure of Tripos molecule structure format (MOL2) file of active ingredients from TCMSP platform were obtained. Additionally, AutoDock tools were also utilized to process the above-mentioned protein receptors and ligands, whereas its plugin AutoGrid was used to detect the docking active sites, and a molecular docking process was conducted to obtain the affinity. In our study, the binding energy ≤ −5.0 kJ · mol^−1^ was used as the key molecule possessing a better binding affinity with the key targets [33].

### 2.7. Survival Analysis

In order to better understand the influence of core targets on NSCLC prognosis, a precise analysis of the influence of the ten main targets on the prognosis of all squamous cell carcinomas and adenocarcinomas of NSCLC from TCGA database (https://www.cancer.gov/about-nci/organization/ccg/research/structural-genomics/tcga) was carried out. The target gene expression was ranked from high to low and used high (50%) and low (50%) cutoff values as expression thresholds to divide the high expression group and the low expression group. The resultant worst prognosis of the high expression group relatively reflected that this specific gene might promote tumor development, and vice versa was considered as a protective factor. Data analysis was conducted on R software, and log-rank test and Kaplan-Meier method were utilized to analyze 1014 samples to obtain survival maps; *p* < 0.05 was considered as a statistically significant difference. Moreover, R software was also used to draw the Sankey diagram in which the width of the extended branch in the figure corresponded to the data flow size, which could be further used to depict the relationship between a certain gene's clinical characteristics, such as smoking, radiation, and racial predilection in NSCLC tumor samples and patient survival, as well as the distribution trend of high and low expressions.

### 2.8. Expression of Core Target Genes in NSCLC Tumor Cell Lines

Since NSCLC has many cell lines, the core genes are distributed differently in various cell lines. For understanding the expression of core genes in the same tumor, we used the Cancer Cell Line Encyclopedia (CCLE) database (https://sites.broadinstitute. org/ccle) to obtain the cell line gene expression matrix of NSCLC tumors and analyzed the expression of core target genes in different NSCLC tumor cell lines by R software, to select suitable cell lines for further verification.

## 3. Results

### 3.1. The Active Ingredients and Chemical Structure of DBD

According to the screening conditions of active ingredients, after obtaining 22 active DBD ingredients from the TCMSP database 182 related key targets were acquired. Since the chemical structures of all active ingredients were collected through the TCMSP database, their MOL2 files were also downloaded by the TCMSP database, which is shown as detailed information in Table 1.

### 3.2. The NSCLC-Related Targets

**Figure 2:**
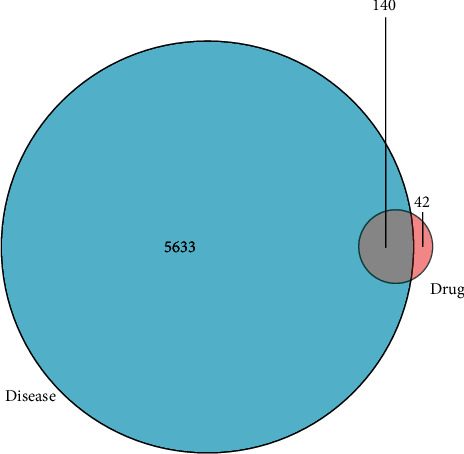
Venn diagram.

The NSCLC-related targets were obtained through GeneCards and OMIM database. In the GeneCards database, 5404 related targets of NSCLC were received, while 468 related targets of NSCLC were gained from the OMIM database. After the merger of two datasets, deduplication was performed, and 5773 related targets of NSCLC were obtained. Additionally, the intersection of the DBD active ingredient targets Venn diagram and the obtained targets of NSCLC through R software yielded 140 core targets. The results are displayed in Figure 2.

### 3.3. Protein-Protein Interaction Construction

**Figure 3:**
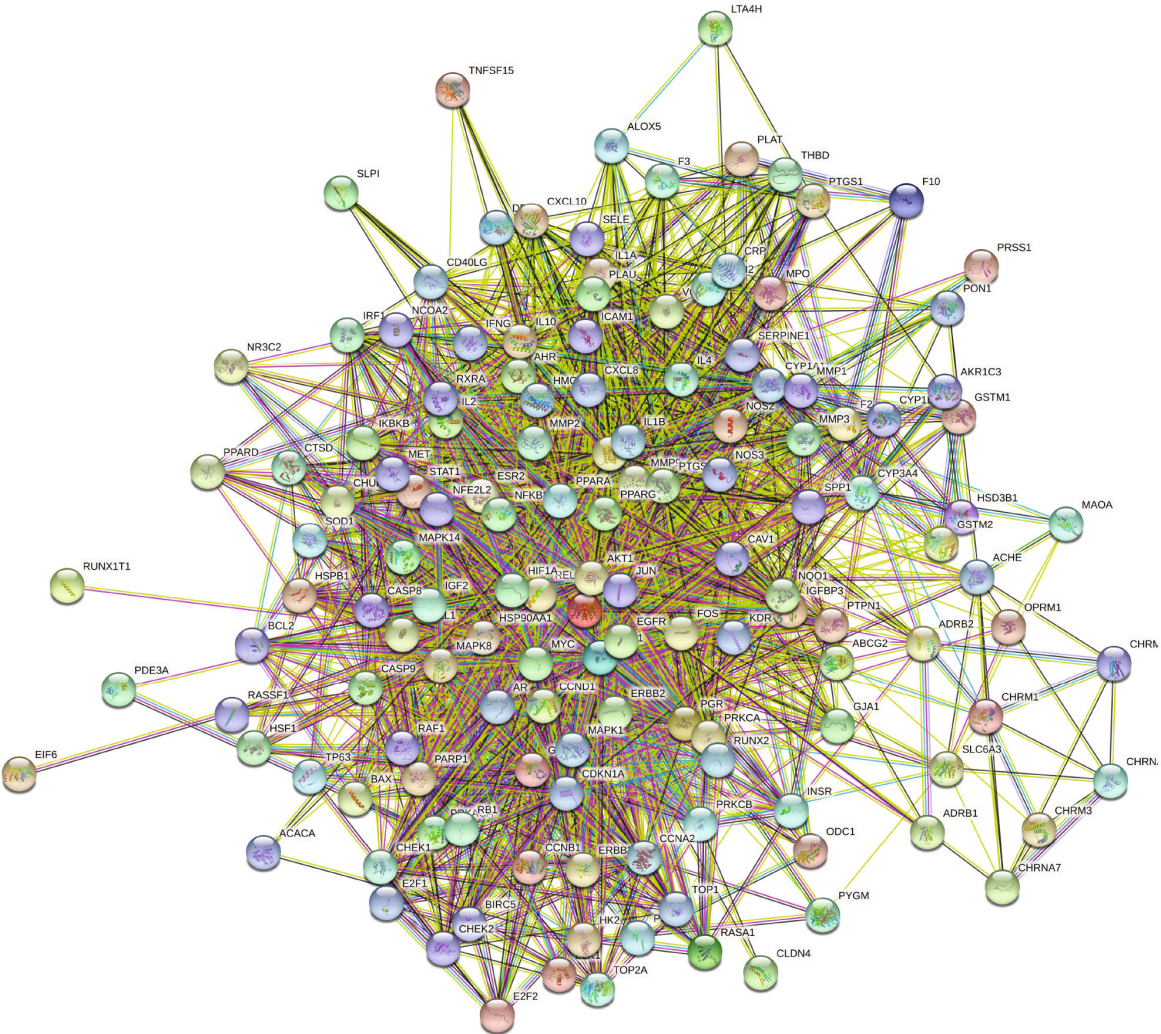
PPI network map.

Several interaction datasets were obtained by incorporating 140 core targets into the string platform and selecting “homo sapiens” to generate the PPI network map, and subsequently, associated protein interaction relationships were acquired. The result is shown in Figure 3. In the PPI network map, there were inherent 140 nodes and 2351 edges in which the nodes represented proteins, edges represented their innate relationships, while the colors from yellow to red represented small to large values.

### 3.4. Drug-Ingredient-Target-Disease Network Construction

**Figure 4:**
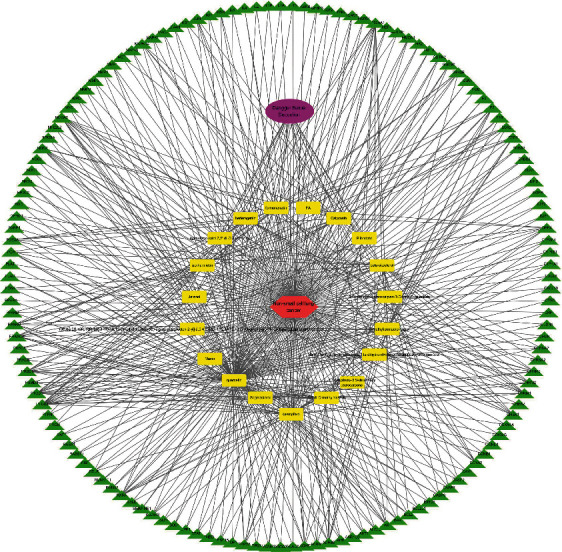
Drug-ingredient-target-disease network diagram.

**Figure 5:**
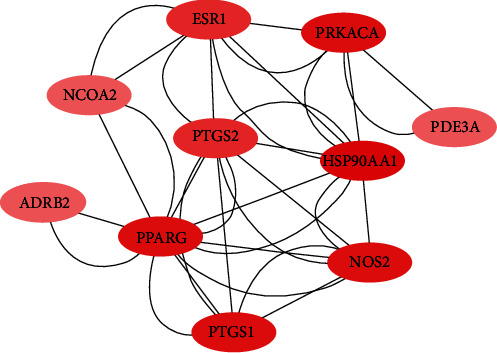
Network diagram between ten main targets.

The drug-ingredient-target-disease network diagram was constructed by Cytoscape software, and the results are displayed in Figure 4. The active ingredients and maximum key targets had numerous interactions through computational inference, thus indicating that DBD can treat NSCLC through a precise analysis of multicomponent, multitarget, and multipathway synergistic interactions, which is quite evident from Figure 5. Using Network Analyzer of Cytoscape software, a degree analysis was conducted, and five main DBD ingredients and ten key targets were screened out. The five main active ingredients were quercetin, kaempferol, formononetin, isorhamnetin, and hederagenin, while the ten core targets were HSP90AA1, NCOA2, PPARG, PRKACA, NOS2, PDE3A, PTGS1, PTGS2, ADRB2, and ESR1. Simultaneously, the Cytoscape software was again utilized to investigate the potential collaborative connections between the ten core targets and thereafter demonstrated the concept that the redder the color, the closer was its connection with other key targets. The result is shown in Figure 5.

### 3.5. GO and KEGG Enrichment Analysis

**Figure 6:**
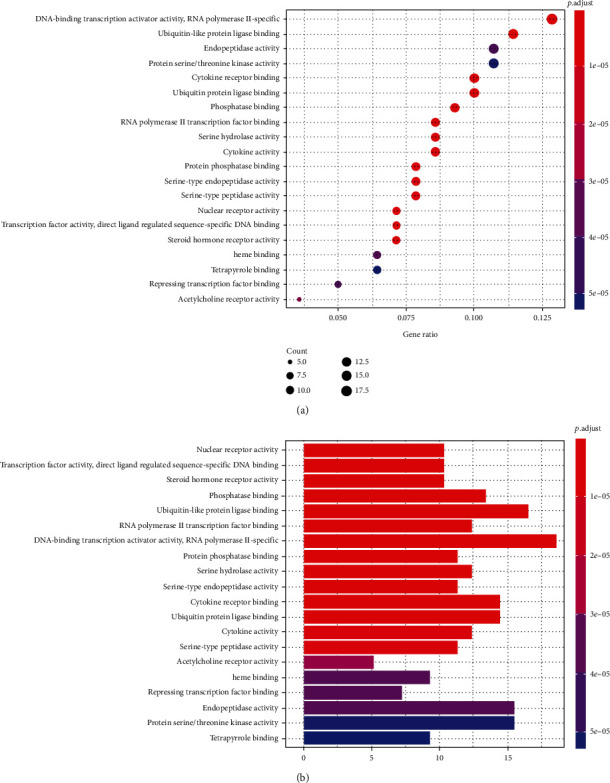
The results of GO functional enrichment analysis. (a) The ordinate includes the biological processes, cell components, and molecular functions involved; the abscissa is the degree of enrichment. The size of the dot represents the number of genes involved; the larger the dot, the greater the number of genes. The lower the *p* value, the redder the color of the graph, and the higher the enrichment degree. (b) The ordinate is the name of the biological processes, cell components, and molecular functions, and the abscissa is the number of genes enriched in each pathway. The *p* value indicates the importance of enrichment; the lower the *p* value, the redder the color of the graph, and the higher the enrichment degree.

Cytoscape and R software were utilized to perform GO functional enrichment analysis on DBD treatment of NSCLC to explore the possible mechanisms of 140 candidate targets for the treatment of NSCLC, and a total of 131 related biological processes, molecular functions, and cellular components were acquired with a significance value of *p* < 0.05. The bubble chart and histogram of the top 20 items are displayed in Figure 6. It was revealed that the coaction targets were mainly enriched in DNA-binding transcription activator activity, RNA polymerase II-specific activity, ubiquitin-like protein ligase binding, endopeptidase activity, protein serine/threonine kinase activity, cytokine receptor binding, ubiquitin-protein ligase binding, etc.

**Figure 7:**
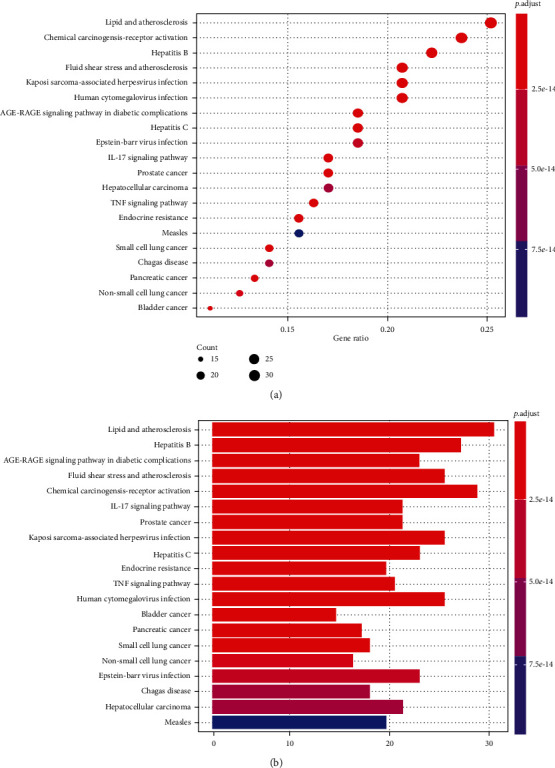
The results of KEGG enrichment analysis. (a) The ordinate includes the related diseases and pathway involved; the abscissa is the degree of enrichment. The size of the dot represents the number of genes involved; the larger the dot, the greater the number of genes. The lower the *p* value, the redder the color of the graph, and the higher the enrichment degree. (b) The ordinate is the name of the related diseases and pathway, and the abscissa is the number of genes enriched in each pathway. The *p* value indicates the importance of enrichment; the lower the *p* value, the redder the color of the graph, and the higher the enrichment degree.

**Figure 8:**
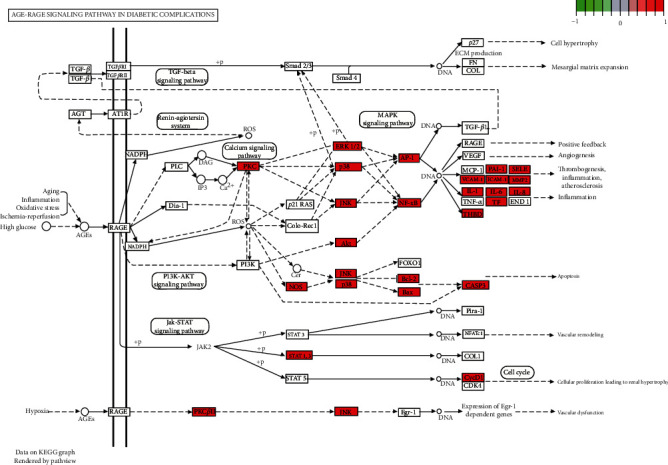
The anti-NSCLC pathway of DBD.

Meanwhile, a similar method was used to perform KEGG enrichment analysis on the DBD treatment regime for NSCLC to explore the possible mechanisms of 140 candidate targets for NSCLC treatment and obtained a total of 174 items with *p* < 0.05. The bubble chart and histogram of the top 20 items are shown in Figure 7. Our study results revealed that the coaction targets were mainly enriched in several diseases: lipid-induced atherosclerosis, hepatitis B, shear stress-induced atherosclerosis, etc., and also participated in different signaling pathways, including the AGE-RAGE signaling pathway in diabetic complications, IL-17 signaling pathway, and TNF signaling pathway.  Figure 8 shows the relevant key targets in both the DBD and AGE-RAGE signaling pathways in diabetic complications.

### 3.6. Molecular Docking

The top five target proteins, as well as the top ten active DBD ingredients in the drug-ingredient-target-disease network, were verified by molecular docking to explore the intertwined interactions between the receptors and ligands. Moreover, if the binding energy was less than zero, it indicated that the ligand molecules could bind to the receptor protein spontaneously while the affinity less than −5.0 kJ·mol^−1^ revealed that the binding affinity was good. Therefore, it can be easily discerned that the smaller the binding affinity, the greater the molecular docking process. Our study results exhibited that all active ingredients bound well to the target proteins, and every affinity was less than −5.0 kJ·mol^−1^ as depicted in Table 2. In particular, quercetin and hederagenin had a better binding affinity to other target proteins. It was also expressed that quercetin possessed an excellent binding affinity to PTGS1 (affinity = −9.7 kJ · mol^−1^) due to the formation of hydrogen bond interactions with the PTGS1 active site, which was the main force promoting its binding capability. Our results also disclosed that these five main active ingredients had better binding affinities towards these protein targets for proper intermolecular interactions.

### 3.7. Survival Analysis

**Figure 9:**
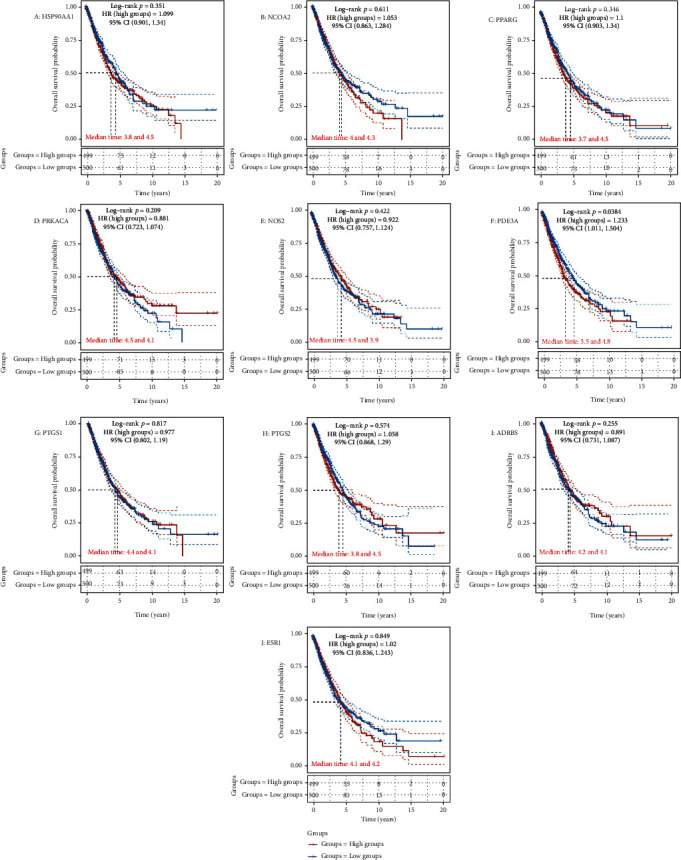
Survival analysis for ten main genes by TCGA.

**Figure 10:**
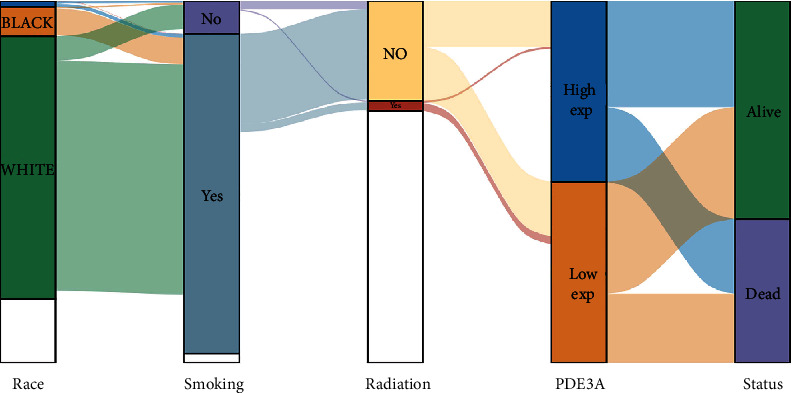
Sankey diagram.

All squamous cell carcinomas and adenocarcinomas of NSCLC cases were categorized into high and low expression groups based on the expression levels of ten core genes, and the correlation between the individual ten core gene's expressions and the prognosis of NSCLC patients was investigated by exploring TCGA database. Our study results revealed that the highly expressed genes were associated with poor prognosis, PDE3A was associated with overall survival in all NSCLC patients (*p* = 0.0384), while the overall survival analysis of other nine core genes with high and low expression did not have any statistical significance (*p* < 0.05) which is shown in Figure 9. As *p* = 0.0384 was utilized to draw the Sankey diagram (Figure 10), the resultant finding unveiled the interwoven relationship between smoking, radiation, racial predilection, the role of PDE3A, and patient survival while showing the potential connection between the width of the extended branch and patient survival.

### 3.8. Expression of Core Target Genes in NSCLC Tumor Cell Lines

**Figure 11:**
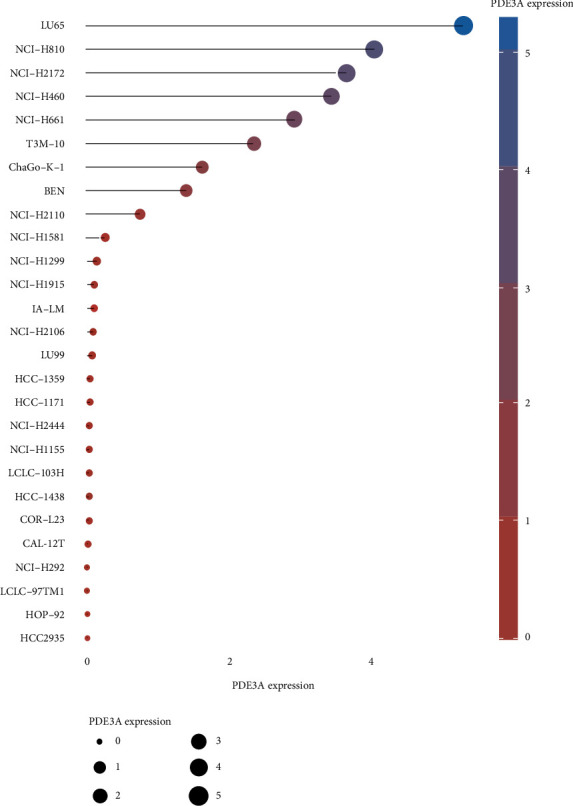
Expression of PDE3A in NSCLC tumor cell lines.

The survival analysis detected that PDE3A had a close connection with patient survival. PDE3A expression in different NSCLC tumor cell lines was analyzed by R software by exploring the CCLE database. Our study results depicted that PDE3A expression was the highest in LU65 and NCI-H810, albeit noting that the abscissa represented the PDE3A expression level while the ordinate represented different NSCLC tumor cell lines, as shown in Figure 11.

## 4. Discussion

While lung cancer is still the leading cause of cancer-related deaths globally, non-small-cell lung cancer (NSCLC) is the most common type of lung cancer, accounting for a large proportion of all lung cancer cases [34, 35]. Therefore, choosing the appropriate medicine integrated with standard oncologic care is particularly important for NSCLC patients. Traditional Chinese medicine is an ancient popular medicine that originated and developed in China. Since it has several unique attributes like the presence of multiple active substances, multiple key targets, and low toxicity [36], it also possesses superior antitumor properties that can be applied in treating different tumors [37]. Our study determined the key active ingredients and possible detailed molecular mechanisms of DBD in the NSCLC treatment through bioinformatics technology to further improve the desired treatment effects as well as to prolong the survival rate of NSCLC patients. In this study, the active ingredients and related targets of DBD were screened from the TCMSP database and further analyzed by GeneCards and OMIM databases to screen out the potential targets for NSCLC. Additionally, the intersection of both DBD ingredient-related targets and the NSCLC-related targets was employed to obtain the core targets. Subsequently, a PPI network and a drug-ingredient-target-disease network were constructed through core targets while using R software later to perform GO function and KEGG pathway enrichment analysis. The top five active DBD ingredients involved in degree analysis were quercetin, kaempferol, formononetin, isorhamnetin, and hederagenin, while the top ten main core targets included HSP90AA1, NCOA2, PPARG, PRKACA, NOS2, PDE3A, PTGS1, PTGS2, ADRB2, and ESR1. Subsequently, the analysis of five main active ingredients with ten core targets by molecular docking process discovered that the combination of quercetin and PTGS1 had the best binding affinity. Additionally, the analysis of ten main target genes survival and the NSCLC prognosis through TCGA database proved that due to the major distribution of PDE3A in the LU65 and NCI-H810 NSCLC tumor cell lines, it has a statistical significance for the prognostic survival time of NSCLC patients,

According to the drug-ingredient-target-disease network, it was observed that DBD has five main active ingredients: quercetin, kaempferol, formononetin, isorhamnetin, and hederagenin. Since quercetin, a bioflavonoid, can induce Hsp70 inhibition involved in growth inhibition of lung cancer cells, it has a great potential as a chemosensitizer in lung cancer treatment as well as the incorporation of dietary quercetin can also be a promising option for cancer prevention [38, 39]. Kuo et al., in their study, verified that kaempferol could be used as a radiosensitizer for NSCLC in vitro and in vivo while significantly improving the lethality of tumor cells [40]. Formononetin is a novel herbal isoflavonoid, which when isolated from herbal medicine might act as a potential chemopreventive drug for lung cancer therapy through induction of cell cycle arrest and apoptosis in NSCLC cells [41]. Isorhamnetin, a traditional Chinese medicine used to treat angina pectoris and acute myocardial infarction, displays a series of antitumor activities [42]. Past literary insights on A549 lung cancer cells discovered that isorhamnetin at a concentration of 20 *μ*g/mL can induce apoptosis in A549 cells, upregulate the expression of apoptotic genes Bax, Caspase-3, and p53, and downregulate the expression of Bcl-2, cyclin D1, and PCNA proteins. Isorhamnetin's mechanism of action may involve apoptosis initiation induced by downregulation of oncogenes and upregulation of apoptotic genes, thus proving that it can significantly inhibit the growth of A549 cells by inducing cell apoptosis [42]. Hederagenin, an oleanolic acid derivative isolated from ivy leaves by displaying potential antitumor activity, might become a promising therapeutic candidate for human colon cancer [43]. A study by Wang et al. reckoned that hederagenin can also induce ROS accumulation and enhance cisplatin and paclitaxel cytotoxicity in lung cancer cells by blocking autophagic flux [44]. Therefore, hederagenin also has a potential synergistic effect in the treatment of lung cancer.

After exploring 5773 candidate NSCLC targets from GeneCards and OMIM databases, 140 common targets were obtained between the NSCLC and DBD, which were subsequently considered as potential targets for the NSCLC treatment. Through the visual analysis of the drug-ingredient-target-disease network and degree ranking, ten core genes were identified. The degree of association between these genes is shown in Figure 6. These ten core genes included HSP90AA1, NCOA2, PPARG, PRKACA, NOS2, PDE3A, PTGS1, PTGS2, ADRB2, and ESR1and may play an important role in tumor cells, particularly in the process of proliferation, migration, and apoptosis. A previous study disclosed that upregulated KCNQ1OT1 levels in NSCLC tissues and cell lines affirmed that higher KCNQ1OT1 levels were related to the poor progression-free survival of NSCLC patients [45]. Additionally, it was found that the HSP90AA1 expression was reduced after downregulating KCNQ1OT1 levels, which proved that KCNQ1OT1 positively regulated the expression of HSP90AA1 by the formation of the miR-27b-3p sponge. These data revealed the role of KCNQ1OT1 as an oncogene through the modulation of the miR-27b-3p/HSP90AA1 axis during the progression of NSCLC. Although it was suggested that HSP90AA1 could be a potential target for NSCLC treatment, another protein-coding gene, NCOA2, might be relevant for gastric cancer, liver cancer, or prostate cancer [46–48]. Although many previous studies have hypothesized that NCOA2 might become a potential therapeutic target for NSCLC, further verification is still needed to validate it [49]. PPARG expression may also act as a potential therapeutic agent for NSCLC, especially for lung squamous cell carcinoma (LSCC), since the activation of PPARG expression can inhibit LSCC development and progress by regulating the upstream regulator and downstream marker genes, which are involved in tumor cell proliferation and protein polyubiquitination/ubiquitination [50]. Another study suggested that the loss of NOS2 reduces the growth of lung tumors and the inflammation caused by oncogenic KRAS while stating that KRAS and NOS2 jointly promote the occurrence and inflammation of lung tumors [51]. Moreover, inhibition of NOS2 may have therapeutic value for lung cancer with oncogenic KRAS mutations. It was also evident that highly methylated DNA, downregulated PDE3A in chemoresistant NSCLC cells by forcing PDE3A expression to make A549/Cis cells sensitive to cisplatin. This result indicated that high PDE3A expression might promote the NSCLC treatment by increased efficacy of combination therapies [52]. A network pharmacology study by Wang et al. on NSCLC did not find any effect of PDE3A on NSCLC [53]. Moreover, in our study, the survival analysis of 10 main targets suggested that only PDE3A had statistical significance for the prognosis of NSCLC patients. The survival rate of PDE3A in the low expression group was higher than that in the high expression group, further confirming that reducing the expression of PDE3A may improve the quality of life of NSCLC patients. Meanwhile, through the Sankey diagram, it was evident that PDE3A was mainly distributed in the LU65 tumor cell line of NSCLC. Interestingly, the LU65 cell line originated in the Asian populations, especially East Asia, accounting for over 79.97% of inhabitants [54]. Therefore, PDE3A might be a more suitable potential therapeutic target for Asian NSCLC patients. Wang et al. analyzed 12 human plasma samples by using RNA-Seq and bioinformatics techniques and found seven key targets related to lung tumorigenesis: COX1, COX2, COX3, ND1, ND2, ND4L, and ATP6 [55]. A previous study demonstrated that the SCC pathogenesis caused by COPD is regulated by HSP90AA1, ADRB2, TBL1XR1, and HSPB1. Therefore, these genes can be used as potential therapeutic targets for the treatment of COPD-related SCC patients [56]. A 2008 study observed that the use of real-time PCR to assess the methylation of the ESR1 promoter in the blood proved very useful for the diagnosis of lung cancer, as these methylated genes might become crucial biomarkers for the early detection of lung cancer. The results also indicated that a comparative evaluation of methylation ratios before and after surgery might be a powerful tool for predicting the prognosis of lung cancer patients [57]. Simultaneously, it was suggested that ESR1 mRNA overexpression is innately associated with NSCLC prognosis [58]. Therefore, ESR1 could also become an important key target for treating NSCLC, which is consistent with the results of Wang et al. [53]. It was also estimated that as PRKACA was a tumor target, it might be helpful as therapy, but due to the lack of evidence, its involvement in the development of lung cancer is debatable.

GO annotation is an important means to examine the function of gene products [59]. Through GO functional enrichment analysis, this study inferred all the important biological processes, their cellular compositions, and intermolecular functions involved in the core targets. The details are displayed in Figure 7. In KEGG enrichment analysis, the results were mainly related to the AGE-RAGE signaling pathway in diabetic complications. Accumulation of AGEs and upregulated expression of RAGE is associated with various pathological conditions, including diabetes, cardiovascular diseases, neurodegenerative disorders, and cancer. The role of AGE-RAGE signaling has been previously demonstrated in the progression of various types of cancer and other pathological disorders [60]. Therefore, the regulation of the AGE-RAGE signaling pathway is closely related to tumor development. In the molecular docking process, analysis of five main active ingredients with ten key core targets while calculating the affinity revealed that the affinity of quercetin and PTGS1 was the greatest (affinity = −9.7 kJ · mol^−1^), while the binding energy of hederagenin and PDE3A was also good (affinity = −8.9 kJ · mol^−1^). These results observed that, although PTGS1 and PDE3A might be the key targets in DBD for treating NSCLC, quercetin and hederagenin may also be effective ingredients for the potential treatment of NSCLC.

However, it should be noted that our study was based on public databases, which had finite information and need to be continuously improved. Additionally, our study also overlooked the influence of concentration, picking time, processing method, and medication time of DBD on NSCLC. But overall, our study is worthy of further exploration and verification to extract the intricate molecular mechanisms governing therapeutic targets for NSCLC treatment.

## 5. Conclusions

Through several bioinformatics methods, our study results predicted the key active ingredients and related targets of DBD as well as explored the potential targets of NSCLC to devise effective drug therapies for the same. Through the enrichment analysis, it was revealed that the potential working mechanism of DBD in NSCLC treatment mainly focused on three crucial interactions: DNA-binding transcription activator activity, RNA polymerase II-specific activity, and chemical carcinogenesis-receptor activation activity. Additionally, few important observations were also discovered. Namely, the AGE-RAGE signaling pathway in diabetic complications, IL-17 signaling pathway, and TNF signaling pathway are the three main DBD signaling pathways, and PDE3A and PTGS1 are the potential key genes, while quercetin and hederagenin are the two main active ingredients crucial for NSCLC treatment. Our study results also depicted that DBD can achieve the goal of treating NSCLC while improving the prognosis of NSCLC patients through a synergistic interplay between multiple ingredients, multiple targets, and multiple signaling pathways. Nevertheless, further experimental validation is also required to ascertain effective therapeutic approaches for NSCLC patients. At a later stage, our team might conduct a following pharmacological study on important signaling pathways and targets of DBD against NSCLC and try to provide a functional basis and rational references toward a new future for drug development and targeted therapy.

## Figures and Tables

**Table 1:** Information of active ingredients and related targets from DBD.

Herb name	Mol ID	Mol name	Mol structure	Related targets	OB (%)	DL
Danggui	MOL000358	Beta-sitosterol	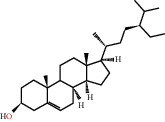	PGR, NCOA2, PTGS1, PTGS2, HSP90AA1, KCNH2, PRKACA, DRD1, CHRM3, CHRM1, SCN5A, CHRM4, PDE3A, ADRA1A, CHRM2, ADRA1B, ADRB2, CHRNA2, SLC6A4, OPRM1, CHRNA7, BCL2, BAX, CASP9, JUN, CASP3, CASP8, PRKCA, PON1, MAP2	36.91	0.75
	MOL000449	Stigmasterol	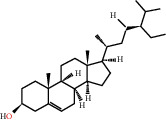	PGR, NR3C2, NCOA2, RXRA, NCOA1, PTGS1, PTGS2, ADRA2A, SLC6A2, SLC6A3, ADRB2, AKR1B1, PLAU, LTA4H, MAOB, MAOA, PRKACA, CTRB1, CHRM3, CHRM1, ADRB1, SCN5A, ADRA1A, CHRM2, ADRA1B, CHRNA7	43.83	0.75
Huangqi	MOL000211	Mairin	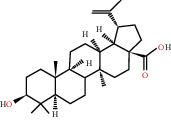	PGR	55.38	0.78
	MOL000239	Jaranol	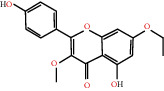	NOS2, PTGS1, AR, SCN5A, PTGS2, ESR2, DPP4, HSP90AA1, CHEK1, PRSS1, NCOA2	50.83	0.29
	MOL000296	Hederagenin	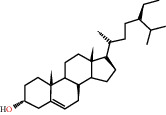	PGR, NCOA2, CHRM3, CHRM1, CHRM2, ADRA1B, GRIA2, LYZ, PTGS1, SCN5A, PTGS2, RXRA, PDE3A, SLC6A2	36.91	0.75
	MOL000033	(3S,8S,9S,10R,13R,14S,17R)-10,13-Dimethyl-17-[(2R,5S)-5-propan-2-yloctan-2-yl]-2,3,4,7,8,9,11,12,14,15,16,17-dodecahydro-1H-cyclopenta[a]phenanthren-3-ol	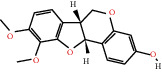	PGR	36.23	0.78
	MOL000354	Isorhamnetin	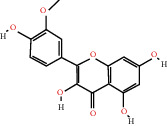	NOS2, PTGS1, ESR1, AR, PPARG, PTGS2, PTPN1, ESR2, DPP4, MAPK14, GSK3B, HSP90AA1, PRKACA, PRSS1, CCNA2, NCOA2, PYGM, PPARD, CHEK1, AKR1B1, NCOA1, F7, F2, ACHE, MAOB, GRIA2, RELA, NCF1, OLR1	49.60	0.31
	MOL000371	3,9-Di-O-methylnissolin	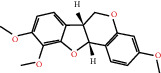	NOS2, PTGS1, CHRM3, F2, CHRM1, ESR1, ADRB1, SCN5A, PTGS2, ADRA2C, RXRA, ACHE, PDE3A, ADRA1B, ADRB2, ADRA1D, OPRM1, PRSS1, NCOA2	53.74	0.48
	MOL000374	5′-Hydroxyiso-muronulatol-2′,5′-di-O-glucoside	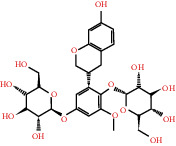	/	41.72	0.69
	MOL000378	7-O-Methylisomucronulatol	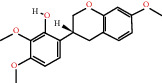	NOS2, PTGS1, DRD1, CHRM3, F2, KCNH2, CHRM1, ESR1, AR, ADRB1, SCN5A, PPARG, F10, CHRM5, PTGS2, ADRA2C, CHRM4, RXRA, OPRD1, PDE3A, ADRA1A, CHRM2, ADRA1B, SLC6A3, ADRB2, ADRA1D, SLC6A4, ESR2, DPP4, MAPK14, GSK3B, HSP90AA1, CHEK1, PRKACA, PRSS1, CCNA2, NCOA2	74.69	0.30
	MOL000379	9,10-Dimethoxypterocarpan-3-O-*β*-D-glucoside	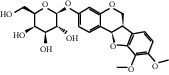	PTGS2, TOP2A, NCOA2	36.74	0.92
	MOL000380	(6aR,11aR)-9,10-Dimethoxy-6a,11a-dihydro-6H-benzofurano[3,2-c]chromen-3-ol	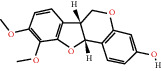	NOS2, PTGS1, CHRM3, F2, CHRM1, ESR1, SCN5A, PTGS2, RXRA, ACHE, ADRA1B, ADRB2, ADRA1D, HSP90AA1, CHRNA7, PRSS1, NCOA2, NCOA1, CHRM4	64.26	0.42
	MOL000387	Bifendate	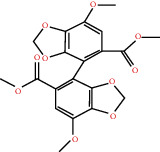	PTGS2, KDR, MET, HSP90AA1, PTGS1, TOP2A	31.10	0.67
	MOL000392	Formononetin	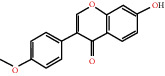	NOS2, PTGS1, CHRM1, ESR1, AR, PPARG, PTGS2, RXRA, PDE3A, ADRA1A, SLC6A3, ADRB2, SLC6A4, ESR2, DPP4, MAPK14, GSK3B, HSP90AA1, MAOB, CHEK1, PRKACA, PRSS1, CCNA2, PKIA, F2, ACHE, JUN, PPARG, IL4, ATP5F1B, ND6, HSD3B2, HSD3B1	69.67	0.21
	MOL000398	Isoflavanone	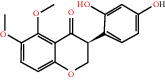	/	109.99	0.30
	MOL000417	Calycosin	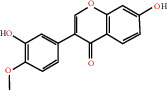	NOS2, PTGS1, ESR1, AR, PPARG, PTGS2, RXRA, PDE3A, ESR2, DPP4, MAPK14, GSK3B, HSP90AA1, CHEK1, PRKACA, PRSS1, CCNA2, NCOA2, ADRB2	47.75	0.24
	MOL000422	Kaempferol	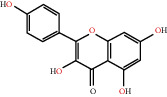	NOS2, PTGS1, AR, PPARG, PTGS2, HSP90AA1, PRKACA, NCOA2, DPP4, PRSS1, PGR, F2, CHRM1, ACHE, SLC6A2, CHRM2, ADRA1B, TOP2A, F7, RELA, IKBKB, AKT1, BCL2, BAX, TNFSF15, JUN, AHSA1, CASP3, MAPK8, MMP1, STAT1, PPARG, HMOX1, CYP3A4, CYP1A1, ICAM1, SELE, VCAM1, NR1I2, CYP1B1, ALOX5, HAS2, AHR, PSMD3, SLC2A4, NR1I3, INSR, DIO1, PPP3CA, GSTM1, GSTM2, AKR1C3, SLPI	41.88	0.24
	MOL000433	FA	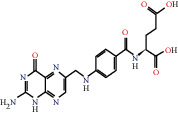	F2, GSK3B	68.96	0.71
	MOL000438	(3R)-3-(2-Hydroxy-3,4-dimethoxyphenyl)chroman-7-ol	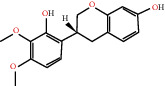	/	67.67	0.26
	MOL000439	Isomucronulatol-7,2′-di-O-glucosiole	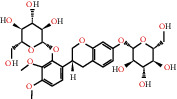	TOP2A	49.28	0.62
	MOL000442	1,7-Dihydroxy-3,9-dimethoxy pterocarpene	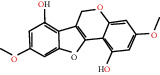	PTGS2, RXRA, HSP90AA1, PRSS1	39.04	0.48
	MOL000098	Quercetin	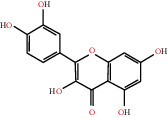	PTGS1, AR, PPARG, PTGS2, F2, HSP90AA1, NCOA2, DPP4, AKR1B1, PRSS1, TOP2A, KCNH2, SCN5A, F10, ADRB2, MMP3, PRKACA, F7, RXRA, ACHE, MAOB, RELA, EGFR, AKT1, CCND1, BCL2, BCL2L1, FOS, CDKN1A, EIF6, BAX, CASP9, PLAU, MMP2, MMP9, MAPK1, IL10, RB1, TNFSF15, JUN, IL6, AHSA1, CASP3, TP63, ELK1, NFKBIA, ODC1, CASP8, TOP1, RAF1, SOD1, PRKCA, MMP1, HIF1A, STAT1, RUNX1T1, ERBB2, PPARG, ACACA, HMOX1, CYP3A4, CAV1, MYC, F3, GJA1, CYP1A1, ICAM1, IL1B, SELE, VCAM1, CXCL8, PRKCB, BIRC5, DUOX2, NOS3, HSPB1, MGAM, IL2, NR1I2, CYP1B1, CCNB1, PLAT, THBD, SERPINE1, IFNG, ALOX5, IL1A, MPO, TOP2A, NCF1, ABCG2, HAS2, NFE2L2, NQO1, PARP1, AHR, PSMD3, SLC2A4, COL3A1, CXCL11, CXCL2, DCAF5, NR1I3, CHEK2, INSR, CLDN4, PPARA, PPARD, HSF1, CRP, CXCL10, CHUK, SPP1, RUNX2, RASSF1, E2F1, E2F2, ACPP, CTSD, IGFBP3, IGF2, CD40LG, IRF1, ERBB3, PON1, DIO1, PCOLCE, NPEPPS, HK2, RASA1, GSTM1, GSTM2	46.43	0.28

**Table 2:** Molecular docking results of 5 main active ingredients and 10 core targets.

Target	PDB ID	Target structure	Active ingredients	Affinity (kJ·mol^−1^)	Best-docked complex
HSP90AA1	3O0I	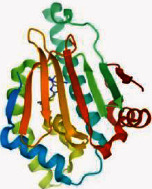	Quercetin	-5.6	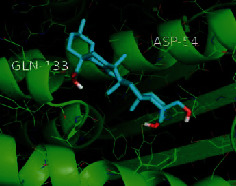
			Kaempferol	-5.4	
			Formononetin	-5.1	
			Isorhamnetin	-5.4	
			Hederagenin	-5.9	

NCOA2	5EHJ	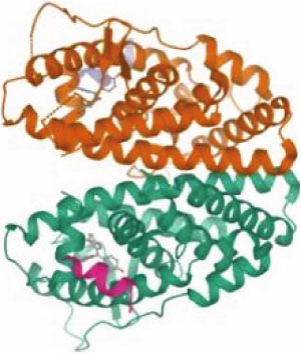	Quercetin	-5.5	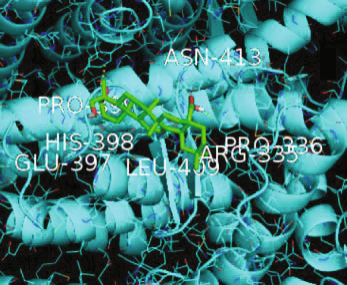
			Kaempferol	-5.3	
			Formononetin	-4.5	
			Isorhamnetin	-5.2	
			Hederagenin	-5.7	

PPARG	6TSG	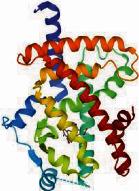	Quercetin	-7.9	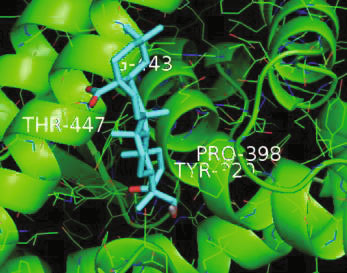
			Kaempferol	-7.3	
			Formononetin	-6.7	
			Isorhamnetin	-7.3	
			Hederagenin	-8.2	

PRKACA	6ZN0	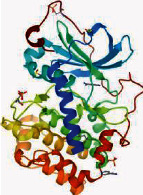	Quercetin	-7.4	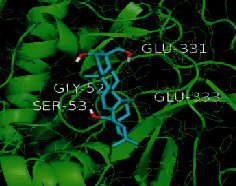
			Kaempferol	-7.1	
			Formononetin	-6.4	
			Isorhamnetin	-7.6	
			Hederagenin	-7.9	

NOS2	1NOS	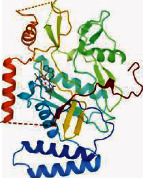	Quercetin	-7.4	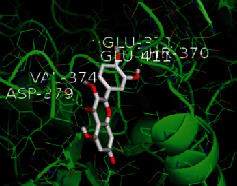
			Kaempferol	-6.9	
			Formononetin	-6.5	
			Isorhamnetin	-6.9	
			Hederagenin	-6.8	

PDE3A	7LRE	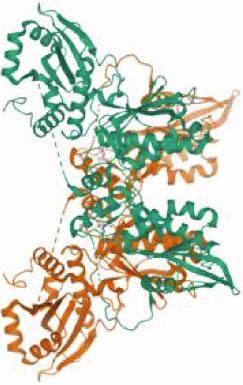	Quercetin	-8.3	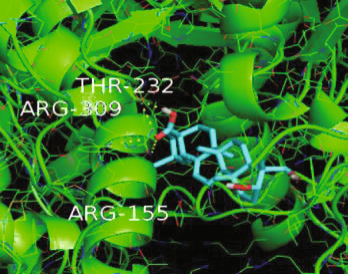
			Kaempferol	-8.4	
			Formononetin	-7.5	
			Isorhamnetin	-8.4	
			Hederagenin	-8.9	

PTGS1	1EBV	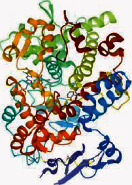	Quercetin	-9.7	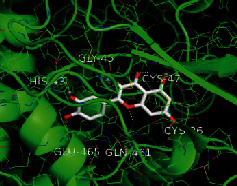
			Kaempferol	-9.6	
			Formononetin	-9.3	
			Isorhamnetin	-9.5	
			Hederagenin	-8.2	

PTGS2	4RUT	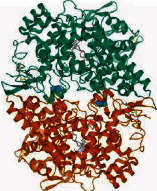	Quercetin	-7.9	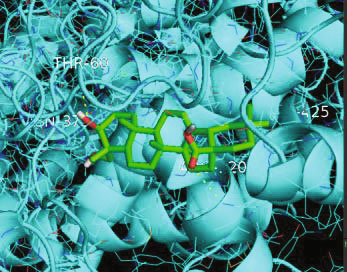
			Kaempferol	-7.7	
			Formononetin	-7.7	
			Isorhamnetin	-7.8	
			Hederagenin	-8.8	

ADRB2	3NY9	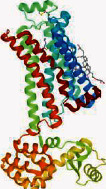	Quercetin	-7.8	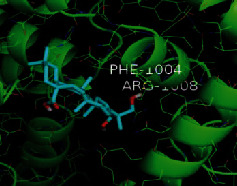
			Kaempferol	-7.8	
			Formononetin	-8.1	
			Isorhamnetin	-7.8	
			Hederagenin	-8.5	

ESR1	6KN5	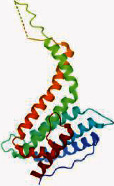	Quercetin	-6.6	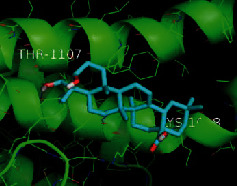
			Kaempferol	-6.7	
			Formononetin	-7.0	
			Isorhamnetin	-6.6	
			Hederagenin	-7.1	

## Data Availability

The data used in this research were from open public databases, but the datasets generated and analyzed in this research can be obtained from the corresponding author on request.

## Abbreviations

CCLE: Cancer Cell Line Encyclopedia
DBD: Danggui Buxue Decoction
DL: Druglikeness
GO: Gene Ontology
KEGG: Kyoto Encyclopedia of Genes and Genomes
NSCLC: Non-small-cell lung cancer
OB: Oral bioavailability
OMIM: Online Mendelian Inheritance in Man
PPI: Protein-protein interaction
TCM: Traditional Chinese medicine
TCMSP: Traditional Chinese medicine systems pharmacology
TCGA: The Cancer Genome Atlas.
